# Response to “Variability of cognitive changes after donanemab treatment”

**DOI:** 10.1002/alz.14520

**Published:** 2025-01-08

**Authors:** Timothy Daly, Kasper P. Kepp, Bruno P. Imbimbo

**Affiliations:** ^1^ UMR 1219 Bordeaux Population Health Université de Bordeaux & INSERM Bordeaux France; ^2^ Bioethics Program FLACSO Argentina Buenos Aires Argentina; ^3^ Epistudia GmbH Bern Switzerland; ^4^ Research & Development Chiesi Farmaceutici Parma Italy

1

The insightful letter^1^ by Dr. Dampney, replying to our recent article on lecanemab and donanemab as disease‐modifying therapies in early Alzheimer's disease (AD),^2^ rightly draws attention to the heterogeneous effect of donanemab on cognitive decline. His analysis has inspired us to perform our own further statistical analysis on the variability of donanemab and lecanemab.

His analysis of donanemab's effect using the Integrated Alzheimer's Disease Rating Scale (iADRS) for individual patients suggests that only ≈60% of patients will experience delays in cognitive decline relative to the mean rate in the placebo group,^1^ not far from a coin toss. The same considerations made for the iADRS can be extended to the Mini‐Mental State Examination (MMSE) for donanemab and the Alzheimer's Disease Assessment Scale‐Cognitive Subscale (ADAS‐Cog) for lecanemab, both widely recognized and objective cognitive scales used in dementia care and research.

We calculated the coefficient of variation (CV) for these two treatments, a standardized measure of dispersion in a probability or frequency distribution that quantifies the extent of individual variability compared to the population mean. It is defined as the ratio of the standard deviation (*σ*) to the mean (*μ*) and is typically expressed as a percentage relative to the mean (Table 1).

**TABLE 1:** Group and individual variability in cognitive response to lecanemab and donanemab in pivotal 18‐month, double‐blind, placebo‐controlled studies in early Alzheimer's disease.

Treatment	Scale	Mean 18‐month group difference	Lower 95% CI value of mean group difference	Higher 95% CI value of mean group difference	Standard error of mean group difference	Drug group (*N* =)	Placebo group (*N* =)	Total sample size	Standard deviation of individual response	Coefficient of variation of individual response
Lecanemab	ADAS‐Cog	−1.44	−2.27	−0.61	0.42	854	872	1726	17.59	1222
Donanemab	MMSE	0.48	0.09	0.87	0.20	429	465	894	5.95	1239

*Source*: Sims et al. (2023)^3^ and van Dyck et al. (2023)^4^. Both studies assumed a normal distribution for most efficacy variables (including the primary outcome measures of efficacy) and presented the data as mean and SD, SEM, or 95% CI.

Abbreviations: ADAS‐Cog, Alzheimer's Disease Assessment Scale‐Cognitive Subscale; CI, confidence interval; SEM, standard error of the mean; SD, standard deviation.

The average slowing effect of donanemab using MMSE compared to placebo was 0.48 on a 30‐point scale at Month 18.^3^ For treatment with lecanemab, the mean group difference between treatment and placebo was −1.44 on a 90‐point scale.^4^ The CV of donanaemab on MMSE (1239%) was similar to lecanemab's effects for ADAS‐Cog at Month 18 (1222%). Thus, for both antibodies, the analysis indicates that the standard deviation of the individual response is 12 times higher than the expected response.

This means that the mean therapeutic benefit of the antibodies is low compared to the individual variability in response. These results, as Dr. Dampney correctly notes, touch on an important point, the major uncertainty in identifying the patient groups likely to benefit. The recent European Medicines Agency (EMA) approval for lecanemab in early AD stipulated that it be used only in apolipoprotein E (*APOE*) ε4 non‐carriers or heterozygotes due to safety concerns related to amyloid‐related imaging abnormalities (ARIA) in homozygotes.^5^ The U.S. Food and Drug Administration (FDA) label for its own approval of lecanemab similarly contains a black box warning due to ARIA.^6^ Dr. Dampney's analysis illustrates that uncertainty is also a major issue for efficacy, and such uncertainty complicates mass administration of a drug, as many patients may experience net harm, especially if one adds to the analysis the adverse effects. The heterogeneity highlighted by Dr. Dampney also complicates the cost–benefit relationship substantially. We consider that further research into the variability of treatment efficacy and adverse effects of anti‐amyloid antibodies in AD is warranted, particularly given discrepancies between the cognitive scales used and the possibility of unblinding effects on efficacy estimations.^7^

In conclusion, the similar variability and apparent efficacy of these two drugs across two separate studies with different sample sizes and cognitive scales is noteworthy. The expectations of individual patients for slowing cognitive decline after anti‐amyloid antibody treatment must contend with significant response variability. These additional important considerations reaffirm our conclusion on the need for rigorous evaluation of the clinical meaningfulness of anti‐amyloid antibodies in AD alongside statistical significance.

## CONFLICT OF INTEREST STATEMENT

Drs. Timothy Daly and Kasper P. Kepp have no conflicts of interest to declare. Dr. Bruno P. Imbimbo is an employee at Chiesi Farmaceutici. He is listed as an inventor in a number of Chiesi Farmaceutici's patents of anti‐Alzheimer drugs. Author disclosures are available in the Supporting Information.

## Supporting information



Supporting Information
